# Viability of Veterinary-Relevant Viruses in Decomposing Tissues over a 90-Day Period Using an In-Vitro System

**DOI:** 10.3390/pathogens12091104

**Published:** 2023-08-29

**Authors:** Ingryd Merchioratto, Cristina Mendes Peter, Akhilesh Ramachandran, Mayara Fernanda Maggioli, Fernando Vicosa Bauermann

**Affiliations:** 1 Department of Veterinary Pathobiology, College of Veterinary Medicine, Oklahoma State University (OSU), Stillwater, OK 74078, USA; 2 Center for Medical Bioinformatics, Escola Paulista de Medicina, Federal University of Sao Paulo (UNIFESP), Sao Paulo 04039-032, SP, Brazil; 3 Molecular Diagnostics, Oklahoma Animal Disease Diagnostic Laboratory, Oklahoma State University (OSU), Stillwater, OK 74078, USA

**Keywords:** bovine, infectivity, inactivation, swine, virus

## Abstract

Depopulation is frequently employed during outbreaks of high-impact animal diseases. Security breaches in sites managing mortality may jeopardize pathogen control efforts as infected carcasses can serve as an infection source. This study evaluated the viability and nucleic acid detection of veterinary-relevant viruses or their surrogates in decomposing tissues. The used viruses were: Senecavirus A1 (SVA), feline calicivirus (FCV), bovine viral diarrhea virus (BVDV), porcine epidemic diarrhea virus (PEDV), bovine alphaherpesvirus 1 (BoHV-1), and swinepox virus (SwPV). Viruses were spiked in three decomposing tissues (swine bone marrow and spleen, and bovine bone marrow) and maintained for 90 days. Samples were kept under two temperature conditions resembling the average soil temperature in central Oklahoma, US, during the winter and summer (5.5 °C and 29.4 °C). At 5.5 °C, SVA and FCV remained viable over the 90 days of the study, followed by BVDV (75 days), BoHV-1 and SwPV (60 days), and PEDV (10 days). At 29.4 °C, SVA remained viable for 45 days, followed by BVDV and BoHV-1 (14 days). SwPV was viable for 10 days, whereas FCV and PEDV were viable for 5 days. Overall, viral nucleic acid detection was not significantly altered during the study. These findings support decision-making and risk management in sites overseeing animal mortality.

## 1. Introduction

In 2021, the agricultural sector in the US, which includes agriculture, food, and related industries, contributed 5.4% to the country’s gross domestic product and represented over 10% of employment in the US [1,2,3]. However, the potential for outbreaks of foreign animal diseases (FADs) poses a serious threat to the US economy and food supply security [2,3,4,5]. Several studies have estimated the economic losses that could result from FAD outbreaks, such as foot-and-mouth disease (FMD), which could range from USD 2.5 million to USD 200 billion [1,2]. FAD outbreaks would impact national and international trade, with added losses related to the costs of eradication, compensation, carcass disposal, cleaning, and decontamination [2].

To prevent FAD outbreaks or limit their spread, effective measures must be taken, including depopulation and proper disposal of carcasses [1,2,5]. Various methods are available for the large-scale disposal of contaminated carcasses. Regardless of the chosen method, certain factors must be considered to ensure the effective handling of these carcasses. These factors include the containment of pathogens to prevent disease spread and environmental protection to safeguard drinking water, air quality, and soil health [6]. For instance, carcass rendering is a safe method for pathogen inactivation. Still transporting infected carcasses outside the outbreak area is concerning [7]. In terms of on-site methods, burning is a safe process to inactivate viruses quickly. However, emissions and pollutants generated during the burning process negatively impact the environment. Another option is deep burial, with a notable risk of groundwater contamination [6].

Alternatives with lower environmental risks include composting and above-ground burial (AGB) methods [6]. Composting may effectively inactivate pathogens through the thermophilic process [6,8]. However, the process requires considerable carbon resources, and the thermophilic process may be affected during the winter months due to colder temperatures [6,8,9,10]. The AGB method is a relatively new method approved for emergency use in the US for large-scale depopulation [6,11]. In this method, carcasses are placed in a shallow grave over a layer of carbon source and covered with a thin (30–40 cm) layer of soil [6,11], minimizing the risk of groundwater contamination compared to deep burial and promoting faster aerobic decomposition [6]. Despite greatly reducing environmental risks, there are certain drawbacks concerning the biosecurity of infected carcasses and tissues during composting and AGB carcass disposal. One common concern is the potential access of scavengers to contaminated tissues. Additionally, extreme weather conditions may compromise the biosecurity measures in place. These factors may pose a risk of pathogen spread [6].

The virus inactivation efficiency of climate-dependent disposal methods varies. Consequently, there is a need to assess the viability of different viruses in decomposing tissues over time under different temperatures. This study evaluated the viability of six viruses spiked in decomposing tissues under two temperature conditions for 90 days. Viral nucleic acid quantification was also performed to assess the level of nucleic acid degradation during the study period and to evaluate the performance of diagnostic protocols.

The viruses used in the study are important livestock pathogens or surrogates of relevant veterinary pathogens and included: Senecavirus A (SVA), feline calicivirus (FCV), bovine viral diarrhea virus (BVDV), bovine alphaherpesvirus 1 (BoHV-1), porcine epidemic diarrhea virus (PEDV), and swinepox virus (SwPV).

## 2. Materials and Methods

### 2.1. Viruses and Cells

The viruses and cell lines used in the study are described in Table 1. Cells were maintained in minimal essential medium (MEM) (Corning^®^, Mediatech, Inc., Manassas, VA, USA) and supplemented with 10% fetal bovine serum (FBS) (Seradigma^®^, VWR International, LLC, Radnor, PA, USA), 2 mM L-glutamine (Corning^®^), 1% Antimycotic-Antibiotic 100× (Gibco™, Grand Island, NY, USA), and 50 µm/mL of gentamicin (Corning^®^). PEDV was amplified in FBS-free media supplemented with 2 µg/mL of L-1-tosylamido-2-phenylethyl chloromethyl ketone (TPCK) trypsin (Sigma-Aldrich^®^, St. Louis, MO, USA). Cells were incubated at 37 °C and in a 5% CO_2_ environment. Virus stocks were titrated using the Reed and Muench method [12], and the titers were calculated and expressed in a median tissue culture infectious dose per milliliter (TCID_50_/mL).

### 2.2. Study Design

To evaluate the viability of SVA, FCV, BVDV, BoHV-1, PEDV, and SwPV in decomposing tissues, three animal tissues were used: swine spleen and bone marrow (BM-pig) collected from culled sows and bovine bone marrow collected from calves (BM-calf). Bone marrow was chosen because the tissue may be contained for a long period within large bones and may be easily transported by scavengers. The spleen may contain a virus during episodes of viremia. Additionally, both tissues sustain viral replication during infection with part of the viruses included in the study [9,10,13]. Procedures were approved by the Institutional Animal Care and Use Committee, protocol numbers VM19-19 (swine tissues) and VM-19-85 (bovine tissues). Tissues were kept at −80 °C until use.

The six viruses were spiked separately to each of the homogenized tissues (spleen, BM-calf, and BM-pig) in the proportion of 1 mL of virus (titer described in Table 1) for 1 g of tissue. Spiked samples were placed in 15 mL bio-reaction tubes that allow gas exchange with the environment (VWR). Each sample was incubated in duplicate for each of the incubation temperatures of 5.5 °C and 29.4 °C. Duplicates of microtubes containing only 1 mL of the viral suspension for each virus were also included as a control.

The temperatures used to incubate the samples were defined based on the evaluation of the average soil temperature during summer and winter in the central part of the state of Oklahoma, US. Soil temperature data at 10 cm in depth was collected from the Mesonet station located in Stillwater (36°08′50.6″ N 97°17′10.2″ W) between 2005 and 2019. The soil temperature data are available at http://www.mesonet.org/index.php (accessed on 10 August 2019).

Samples were kept for up to 90 days at both temperatures in incubators, and collections were performed on days 0, 5, 10, 14, 20, 25, 31, 45, 60, 75, and day 90. A set of tubes was prepared for each sampling point. For each tube, vortex homogenization was performed, and the liquid portion of the sample was collected and transferred to a sterile microtube and stored at −80 °C until testing. At least 200 µL of sample per tube was recovered. The liquid portion of the samples could be collected up to day 31. The membrane in the lid of the bio-reaction tube allowed for the slow evaporation of liquid content. Thus, after this point, 1 mL of sterile phosphate-buffered saline (PBS) was added to samples collected from day 45 to day 90. The added PBS was homogenized, and the liquid portion of the sample was collected and stored as described above.

### 2.3. Virus Titration and Isolation

Collected samples were centrifuged for 5 min at 1200× *g*, and the supernatant was transferred to a new microtube. Viral titrations were conducted in duplicate for each of the independent duplicate samples. The retrieved titers were used in a linear regression model to estimate virus viability length for the viruses with measurable titers on day 90. The analyses were performed using GraphPad Prism (version 9.2.0).

Samples with titers below the titration assay threshold (10^1.8^ TCID_50_/mL) were submitted to the viral isolation (VI) assay. For this, the samples were diluted 1:5 (100 µL of sample in 400 µL of MEM with 25,000 units/mL of penicillin and 25,000 µg/mL of streptomycin) and inoculated in 24-well plates containing a 70% to 80% confluent cell layer. Up to 5 passages of 3–6 days each were performed. Samples were evaluated for the presence of a cytopathic effect under an inverted light microscope.

### 2.4. Viral Nucleic Quantification

One-step RT-qPCR reactions for SVA, FCV, BVDV, and PEDV or qPCR for BoHV-1 and SwPV were conducted on samples from days 5, 45, and 90 of the experiment to assess potential viral nucleic acid degradation. The Oklahoma Animal Disease Diagnostic Laboratory (OADDL) performed the viral nucleic acid extraction and amplification steps following established operational procedures. Briefly, nucleic acid extractions were performed in the KingFisher™ Flex Magnetic Particle Processors (Thermo Fisher Scientific, Waltham, MA, USA) using the MagMAX Pathogen RNA/DNA kit (Thermo Fisher Scientific) according to the manufacturer’s instructions. The SVA amplification was performed with the Tetracore EZ-SVA Real-Time RT-PCR kit (Tetracore^®^, Rockville, MD, USA) according to the manufacturer’s instructions. For PEDV, the VetMax PEDV/TGEV/SDCoV Real-Time RT-PCR kit (Thermo Fisher Scientific) was used, following the manufacturer’s recommendations. The FCV was amplified using the primer pair FCV-F 5′-GTTGGATGAACTACCCGCCAATC-3′ and FCV-R 5′-CATATGCGGCTCTGATGGCTTGAAACTG-3′, and the FCV probe [6FAM] 5′-TCGGTGTTTGATTTGGCCTG-3′ [TAMRA], as previously described [14].

The BVDV and BoHV-1 amplification employed a multiplex assay using the AgPath Multiplex Enzyme kit (Thermo Fisher Scientific) according to the manufacturer’s instructions. The primers were BVDV-F 5′-GGGNAGTCGTCARTGGTTCG-3′, BVDV-R 5′-GTGCCATGTACAGCAGAGWTTTT-3′, BoHV-F 5′-GGCACTGRGACCCTCGTGTT-3′, and BoHV-R 5′-TTGATCTCGCGGAGGCAGTA-3′. The probes included were BVDV [6FAM] 5′-CCAYGTGGACGAGGGCAYGC-3′ [BQH-1] and BHV [TAMRA] 5′-CCGCGTGCCTCTGCTACCCCTTC-3′ [BHQ-1]. The amplification of SwPV used the primers Pox-F 5′-TCAGTACATCCAATTGTCAAGGA-3′, and Pox-R 5′-CTGGCTAAATAGAATGAGTGAAACG-3′, and the probe [6FAM] 5′-ACTTCCAGAAACGAGTAATCCTTACAAGAC-3′ [BHQ-2] (Integrated DNA Technologies), under the conditions previously described [13].

## 3. Results and Discussion

### 3.1. Virus Viability

#### 3.1.1. SVA

**Figure 1:**
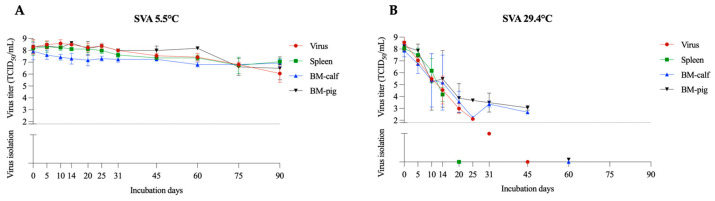
SVA viability in spleen and bone marrow (BM-calf and BM-pig) in decomposing tissues over 90 days. SVA titration and virus isolation results at 5.5 °C (**A**) and 29.4 °C (**B**). The dotted line indicates the detection limit of the titration assay (1.8 TCID_50_/mL). Below the dotted line are presented the virus isolation results, with virus isolation negative samples presented over the X axis.

SVA emerged as an important swine pathogen in 2014 and caused significant outbreaks of vesicular disease clinically indistinguishable from FMDV [15]. SVA and FMDV belong to the *Picornaviridae* family, and SVA had been previously employed as an FMDV surrogate in viability studies [13,16]. SVA demonstrated increased resistance in the tested conditions, remaining viable in all tested tissues for the 90 days of the study at 5.5 °C (Figure 1A). Interestingly, there was a minor decrease in titer over the study period. On day 90 of the experiment, the SVA had a mean titer of 10^7.1^ TCID_50_/mL in the spleen, 10^6.9^ TCID_50_/mL in the BM-calf, and 10^6.5^ TCID_50_/mL in the BM-pig. The linear regression model with 95% confidence interval indicated an expected viability between 403 and 624 days in the spleen, 565 to 1274 days in the BM-calf, and between 336 and 578 days in the BM-pig. These results corroborate findings from previous studies that demonstrated increased viability of SVA in various conditions and temperatures [13,17,18]. The SVA results also align with studies reporting FMDV viability in the bone marrow for up to 7 months when stored at 1–4 °C [19] and 77 days in muscle when stored at 4 °C [20]. Another study demonstrated that FMDV could persist for more than 98 days in slurry and about 8 weeks in minimal essential medium (MEM) [21]. SVA viability was significantly reduced in the samples incubated at 29.4 °C. Positive titration was retrieved until day 45 in BM-calf (average titer of 10^2.7^ TCID_50_/mL) and BM-pig (average titer of 10^3.1^ TCID_50_/mL), and up to day 14 in spleen (average titer of 10^4.2^ TCID_50_/mL) (Figure 1B). At room temperature (20–23 °C), FMDV remained viable for 9 days [21] in MEM, 10 days in skin pool [22], and 14 days in slurry [21]. Despite the decreased viability period in higher temperatures, the length of SVA viability was also significantly affected by the matrix containing the virus, with BM tissue viability at least 26 days longer compared to the spleen samples.

#### 3.1.2. FCV

**Figure 2:**
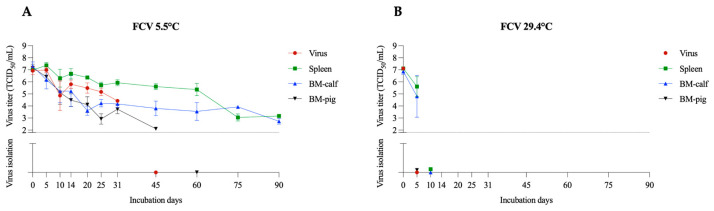
FCV viability in spleen and bone marrow (BM-calf and BM-pig) in decomposing tissues over 90 days. FCV titration and virus isolation results at 5.5 °C (**A**) and 29.4 °C (**B**). The dotted line indicates the detection limit of the titration assay (1.8 TCID_50_/mL). Below the dotted line are presented the virus isolation results, with virus isolation negative samples presented over the X axis.

FCV is a highly prevalent virus in cats and causes upper respiratory tract clinical signs and oral disease [23]. FCV belongs to the *Caliciviridae* family and was used as a surrogate for vesicular exanthema of swine virus (VESV), another member of this family that leads to a vesicular disease resembling FMDV in swine [17]. At a temperature of 5.5 °C, FCV remained viable for the 90 days of the experiment in the spleen and BM-calf samples, with an average titer of 10^3.2^ TCID_50_/mL and 10^2.7^ TCID_50_/mL, respectively (Figure 2A). The linear regression with 95% confidence interval suggests viability between 149 and 185 days in the spleen, whereas between 129 and 205 days in the BM-calf. Notably, the importance of the matrix in virus viability is demonstrated in the FCV samples. In BM-pig, FCV viability was limited to 45 days, with an average titer of 10^2.1^ TCID_50_/mL. It is important to note the decreased fat concentration in the bone marrow of young animals (BM-calf) compared to the bone marrow of adult animals (BM-pig) [24]. The difference in the bone marrow composition may be a possible explanation for the difference in viability.

In samples kept at 29.4 °C, the FCV viability was reduced to 5 days in the spleen and BM-calf (average titer of 10^5.6^ TCID_50_/mL and 10^4.8^ TCID_50_/mL, respectively) (Figure 2B). BM-pig samples were negative in viral titration and isolation on day 5 of the experiment. Previous studies described FCV viability at 4 °C for up to 55 days in MEM [25]. However, the viability was reduced to 7 days in feces at the same temperature [26]. A significant influence of the temperature on the FCV inactivation was also observed in MEM samples at 37 °C, in which viability was limited to 5 days in a previous study [25]. The influence of the temperature on the FCV viability is noteworthy.

#### 3.1.3. BVDV

**Figure 3:**
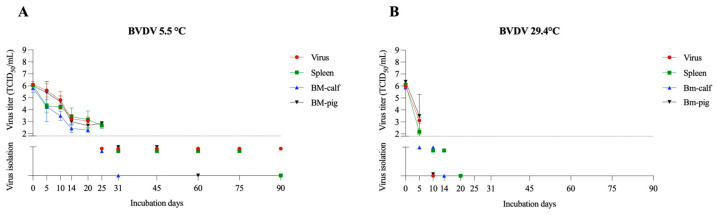
BVDV viability in spleen and bone marrow (BM-calf and BM-pig) in decomposing tissues over 90 days. BVDV titration and virus isolation results at 5.5 °C (**A**) and 29.4 °C (**B**). The dotted line indicates the detection limit of the titration assay (1.8 TCID_50_/mL). Below the dotted line are presented the virus isolation results, with virus isolation negative samples presented over the X axis.

BVDV is associated mainly with respiratory and reproductive syndromes in cattle [27]. BVDV belongs to the same family as CSFV, *Flaviviridae*, and has been used as its surrogate in previous studies [13,17]. At 5.5 °C, BVDV remained viable for up to 75 days in the spleen, followed by 45 days in BM-pig and 25 days in BM-calf based on VI results. However, detectable titers were demonstrated up to day 20 in BM-calf (average titer of 10^2.3^ TCID_50_/mL) and day 25 in BM-pig and spleen (with an average titer of 10^2.9^ and 10^2.7^ TCID_50_/mL, respectively) (Figure 3A). At 4 °C, CSFV stayed viable for 40–60 days in feces and 20–23 days in urine [28], whereas BVDV was reported to be viable for more than 42 days in slurry [21]. In samples incubated at 29.4 °C, BVDV remained viable in the spleen for 14 days, and in BM-calf for 10 days. Detectable titers were seen on day 5 in BM-pig (average titer 10^3.5^ TCID_50_/mL) and spleen (average titer 10^2.2^ TCID_50_/mL). In samples maintained at 29.4 °C, the longest viability period was 14 days (spleen) (Figure 3B). Previously, BVDV was reported viable for up to 20 days at room temperature (21–23 °C) in spiked bone marrow samples [13]. Another study predicted CSFV could remain viable for about 22 weeks in muscle tissue at 4 °C, while at 25 °C, it would remain viable for 24 days [29]. Virus inactivation can be influenced b y lipases [30], which may be present at higher levels in some matrices containing a high fat content [29], which may explain the decreased viability of BVDV in bone marrow samples compared to the spleen. However, the difference in virus viability in bone marrow samples was observed only for BVDV in our study.

#### 3.1.4. PEDV

**Figure 4:**
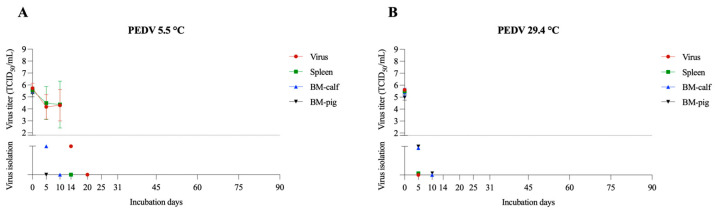
PEDV viability in spleen and bone marrow (BM-calf and BM-pig) in decomposing tissues over 90 days. PEDV titration and virus isolation results at 5.5 °C (**A**) and 29.4 °C (**B**). The dotted line indicates the detection limit of the titration assay (1.8 TCID_50_/mL). Below the dotted line are presented the virus isolation results, with virus isolation negative samples presented over the X axis.

PEDV is a single-stranded RNA virus that belongs to the family *Coronaviridae* and is in the genus *Alphacoronavirus*. The virus is highly transmissible in pigs, causing outbreaks of severe diarrhea and high mortality in young pigs, leading to significant economic impact [31]. PEDV samples kept at 5.5 °C remained viable for a longer time in the spleen (10 days) with an average titer of 10^4.4^ TCID_50_/mL, followed by 5 days in the BM-calf. In the BM-pig samples, no viable virus was identified on day 5 (Figure 4A). At a temperature of 29.4 °C, PEDV only remained viable in BM-calf and -pig for 5 days based on the viral isolation assay (Figure 4B). Some studies have shown that PEDV can remain viable for 3 weeks at 4 °C in spray-dried bovine plasma [32], and in conditions from −20 °C to 4 °C for 28 days [33], whereas in our study, PEDV remained viable for up to 10 days in spleen samples at 5.5 °C. In feces, at temperatures of 20–23 °C, PEDV was viable for up to 14 days [33]. In our study, PEDV, when incubated at 29.4 °C, remained viable for a maximum of 5 days.

#### 3.1.5. BoHV-1

**Figure 5:**
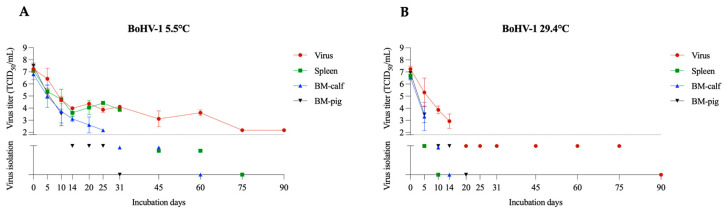
BoHV-1 viability in spleen and bone marrow (BM-calf and BM-pig) in decomposing tissues over 90 days. SVA titration and virus isolation result at 5.5 °C (**A**) and 29.4 °C (**B**). The dotted line indicates the detection limit of the titration assay (1.8 TCID_50_/mL). Below the dotted line are presented the virus isolation results, with virus isolation negative samples presented over the X axis.

BoHV-1 was used as a surrogate for pseudorabies virus (PRV), and as does BoHV-1, it belongs to the *Herpesviridae* family. BoHV-1 is associated with respiratory and reproductive manifestations in cattle worldwide [34]. Our results also showed the important effect of the matrix. At a temperature of 5.5 °C (Figure 5A), BoHV-1 remained viable for 60 days in the spleen, 45 days in the BM-calf, and 25 days in the BM-pig. The spleen and BM-calf samples remained with measurable BoHV-1 titers until day 31 (average titer 10^3.9^ TCID_50_/mL) and day 25 (average titer 10^2.2^ TCID_50_/mL), respectively. In the BM-pig, the average titer of 10^3.5^ TCID_50_/mL was detected on day 10. It was previously reported that at temperatures ranging from 3 to 16 °C, PRV remained viable for 37 days in pork sausage casings [35]. At the temperature of 29.4 °C, BoHV-1 remained viable in the BM-pig for 14 days, and 10 days in the BM-calf. Virus titration yielded positive titers on day 5 in the BM-pig (average titer of 10^3.5^ TCID_50_/mL) and BM-calf (average titer of 10^3.3^ TCID_50_/mL) (Figure 5B). In the spleen samples, BoHV-1 was identified only on day 5, by VI assay. In PRV infected carcasses submitted to composting, no viable virus was detected after 7 days (compost piles reached between 30 and 35 °C) [36].

#### 3.1.6. SwPV

**Figure 6:**
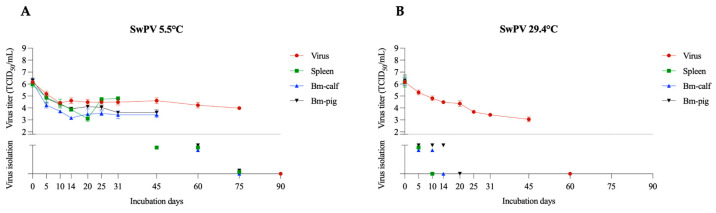
SwPV viability in spleen and bone marrow (BM-calf and BM-pig) in decomposing tissues over 90 days. SVA titration and virus isolation results at 5.5 °C (**A**) and 29.4 °C (**B**). The dotted line indicates the detection limit of the titration assay (1.8 TCID_50_/mL). Below the dotted line are presented the virus isolation results, with virus isolation negative samples presented over the X axis.

SwPV, as well as lumpy skin disease virus (LSDV), belongs to the *Poxviridae* family. Although SwPV has a worldwide distribution, cases of the disease in pigs are rarely reported and include skin vesicles [37]. LSDV has been the focus of attention due to current outbreaks and rapid dissemination [38,39]. The transmission potential of LSDV has been associated with vectors, mainly arthropods [38,39]. Another important characteristic of this family is resistance in the environment, with reports of a closely related virus, contagious ecthyma virus (OrfV), remaining viable in the environment for years [40,41]. Additionally, SwPV has been considered a potential model to study ASFV viability due to its structural similarities [13,42]. At 5.5 °C, SwPV remained detectable by virus isolation for 60 days in the three tested tissues (Figure 6A). Titers were detected in the BM-pig samples on day 45 (average titer of 10^3.6^ TCID_50_/mL) and in the BM-calf (average titer of 10^3.4^ TCID_50_/mL). In spleen samples, an average titer of 10^4.8^ TCID_50_/mL was retrieved on day 31. At a temperature of 29.4 °C, SwPV remained viable in BM-pig for 14 days, followed by BM-calf (10 days) and spleen for 5 days (Figure 6B). SwPV titrations of samples at 29.4 °C were inconclusive due to heavy bacterial contamination and the impossibility of filtering the samples due to the large SwPV virion size.

In a previous study, SwPV remained viable in bone marrow for more than 30 days at room temperature (21–23 °C) and for less than 7 days in bone marrow buried under temperatures of 34–36 °C [13]. In comparison, ASFV remained viable in bone for 30 days at a temperature of 4 °C and only for 7 days at a temperature of 21 °C [43].

### 3.2. Nucleic Acid Extraction and qPCR Assay

**Figure 7:**
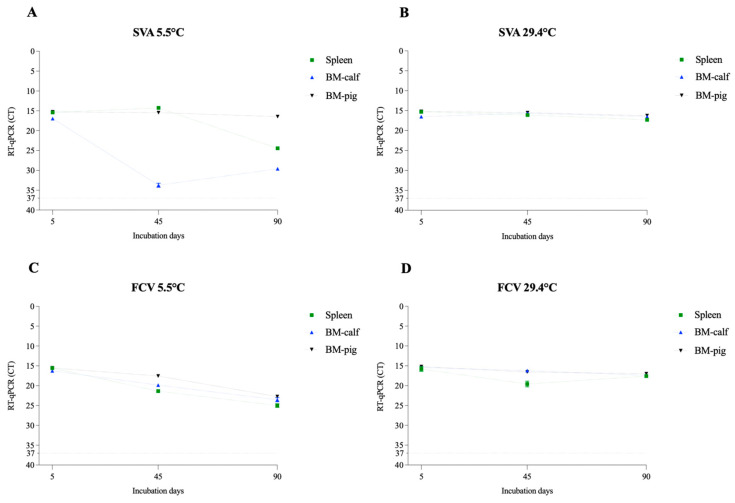
Evaluation of virus nucleic acid detection of the non-enveloped RNA viruses in spleen and bone marrow (BM-calf and BM-pig) in decomposing tissues over a 90-day period. The cycle threshold (CT) values for SVA at 5.5 °C (**A**) and 29.4 °C (**B**) and for the FCV at 5.5 °C (**C**) and 29.4 °C (**D**). The line at mark 37 represents CT 37, considered the threshold limit for detection.

**Figure 8:**
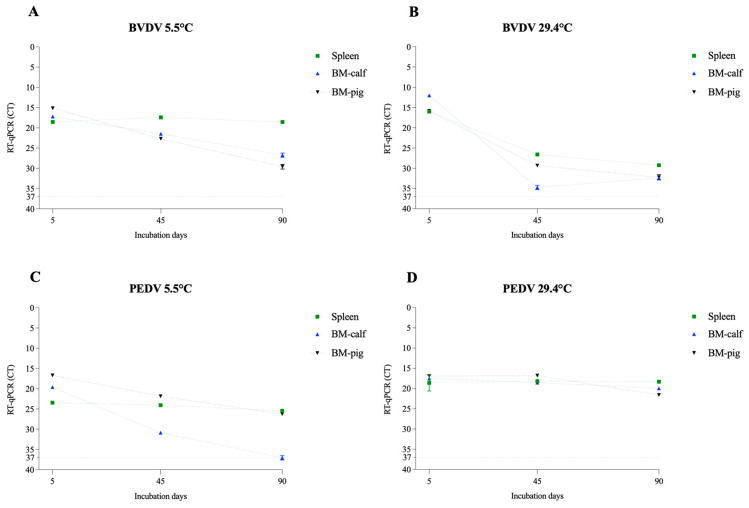
Evaluation of virus nucleic acid detection of the enveloped RNA viruses in spleen and bone marrow (BM-calf and BM-pig) in decomposing tissues over a 90-day period. The cycle threshold (CT) values for BVDV at 5.5 °C (**A**) and 29.4 °C (**B**) and for the PEDV at 5.5 °C (**C**) and 29.4 °C (**D**). The line at mark 37 represents CT 37, considered the threshold limit for detection.

**Figure 9:**
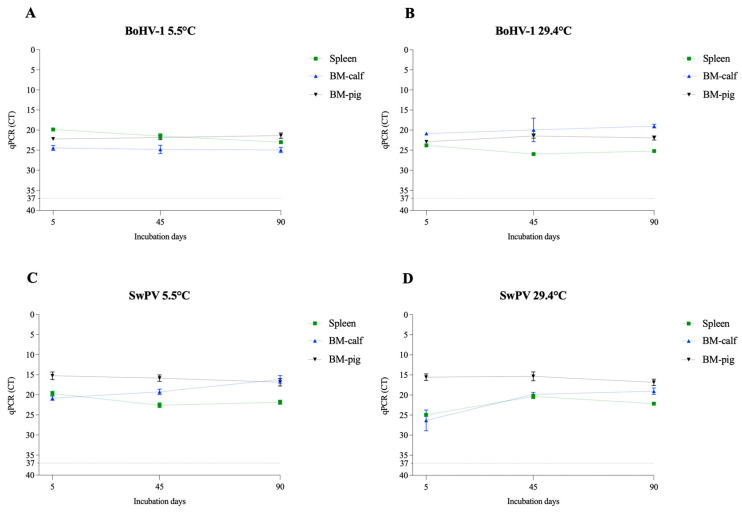
Evaluation of virus nucleic acid detection of the enveloped DNA viruses in spleen and bone marrow (BM-calf and BM-pig) in decomposing tissues over a 90-day period. The cycle threshold (CT) values for BoHV-1 at 5.5 °C (**A**) and 29.4 °C (**B**) and for the SwPV at 5.5 °C (**C**) and 29.4 °C (**D**). The line at mark 37 represents CT 37, considered the threshold limit for detection.

In addition to viability, we performed RT-qPCR and qPCR to evaluate the potential degradation of viral nucleic acid during the study period. The assays were performed on the samples collected on days 0, 45, and 90. All tested samples were positive for specific viral nucleic acid amplification, demonstrating the potential use of standard diagnostic protocols in identifying these pathogens in decomposing tissues for at least 3 months (Figure 7, Figure 8 and Figure 9). With the exception of PEDV, the decrease in the viral nucleic acid concentration was more evident in the enveloped RNA viruses. These results also corroborate previous studies that detected virus nucleic acid in various matrices under different conditions, such as ASFV in spray-dried porcine plasma for 5 weeks at 4 °C [44] and PEDV in manure stored at −30 °C to 23 °C for 9 months [45].

## 4. Conclusions

In situations where security breaches occur at facilities involved in mortality management, infected tissues and carcasses can act as potential sources of viral contamination. Understanding the dynamics of virus inactivation in various tissues is critical to establishing parameters that can assist in decision-making and response strategies to mitigate the spread of highly impactful animal disease outbreaks. The findings of this study demonstrate that the viability of all viruses was significantly longer at a temperature of 5.5 °C, with an average duration of 60 days compared to an average of 14 days at 29.4 °C. The viability results are summarized in Table 2.

Despite the fact that non-enveloped viruses are typically considered more resistant compared to enveloped viruses [46], the data derived from our study reinforce the notion that specific conditions significantly influence the viability of enveloped and non-enveloped viruses. Factors including temperature and matrix must be considered in the event of outbreaks. In addition, the pH (not evaluated in this study) should be considered in future studies as a critical factor potentially affecting virus viability. Our study considered the average soil temperature in central Oklahoma during summer (29.4 °C) and winter (5.5 °C). In contrast, in regions characterized by harsh winter seasons, the inactivation of viruses in carcasses undergoing decomposition in composting or in-ground systems may be extended. To illustrate this point, in Sioux County-IA, one of the largest pork producers in the United States, the average soil temperature at a depth of 10 cm in the winter of 2022 was −2.8 °C and remained below 5.5 °C for approximately 144 days, from November 2022 to April 2023. (Data obtained from Menoset IAstate https://mesonet.agron.iastate.edu/smos/; accessed on 1 July 2023) Consequently, the environmental conditions of each location managing mortality should be taken into consideration for the appropriate security of facilities involved in depopulation events. Likewise, the nature of the pathogen and the influence of the different matrices present in a carcass should be taken into consideration.

## Figures and Tables

**Table 1:** Descriptions of viruses and cell lines used in the study. The viral titers of the stocks used in the experiments are also presented.

Target Virus	Surrogate Viruses Employed	Viral Family	Envelope	Genetic Material	Cell Line	Virus Titer (TCID_50_/mL)
Foot and mouth disease virus (FMDV)	Senecavirus A1 (SVA)	*Picornaviridae*	Non-enveloped	+ssRNA	ST	10^8.8^
Vesicular exanthema of swine virus (VESV)	Feline calicivirus (FCV)	*Caliciviridae*	Non-enveloped	+ssRNA	CRFK	10^8.2^
Classical swine fever virus (CSFV)	Bovine viral diarrhea virus (BVDV)	*Flaviviridae*	Enveloped	+ssRNA	MDBK	10^6.0^
Porcine epidemic diarrhea virus (PEDV)	Not applied	*Coronaviridae*	Enveloped	+ssRNA	VERO	10^6.0^
Pseudorabies virus (PRV)	Bovine alphaherpesvirus 1 (BoHV-1)	*Orthoherpesviridae*	Enveloped	dsDNA	MDBK	10^7.6^
Lumpy skin diseases virus (LSDV); African swine fever virus (ASFV)	Swinepox virus (SwPV)	*Poxviridae*	Enveloped	dsDNA	PK-15	10^6.8^

**Table 2:** Summary of the viability length for each tested virus in spleen and bone marrow tissues (BM-calf and BM-pig).

Viruses	Tissue	Viability (Days) in the Tested Temperatures	
		5.5 °C	29.4 °C
Senecavirus A1 (SVA)	Spleen	≥90 *	14
	BM-calf	≥90	45
	BM-pig	≥90	45
Feline calicivirus (FCV)	Spleen	≥90	5
	BM-calf	≥90	5
	BM-pig	45	<5 **
Bovine viral diarrhea virus (BVDV)	Spleen	75	14
	BM-calf	25	10
	BM-pig	45	5
Porcine epidemic diarrhea virus (PEDV)	Spleen	10	<5
	BM-calf	5	5
	BM-pig	<5	5
Bovine alphaherpesvirus 1 (BoHV-1)	Spleen	60	5
	BM-calf	45	10
	BM-pig	25	14
Swinepox virus (SwPV)	Spleen	60	5
	BM-calf	60	10
	BM-pig	60	14

* Virus viable for 90 days or more. ** Virus viable for less than 5 days.

## Data Availability

Not applicable.
